# Biogenic synthesis of silver nanoparticle by *Cytobacillus firmus* isolated from the river sediment with potential antimicrobial properties against *Edwardsiella tarda*

**DOI:** 10.3389/fmicb.2024.1416411

**Published:** 2024-08-30

**Authors:** Vikash Kumar, Satya Narayan Parida, Souvik Dhar, Kampan Bisai, Dhruba Jyoti Sarkar, Soumya Prasad Panda, Basanta Kumar Das

**Affiliations:** ^1^Biotechnology Laboratory, ICAR-Central Inland Fisheries Research Institute, Barrackpore, India; ^2^College of Fisheries, Rani Lakshmi Bai Central Agricultural University, Jhansi, India

**Keywords:** *Cytobacillus firmus*, silver nanoparticle, FTIR, TEM, antimicrobial properties

## Abstract

The aquatic environment, independent of their host, is more favorable to pathogenic bacteria than the terrestrial environment. Consequently, pathogenic bacteria can reach very high densities around aquatic animals and can cause high mortality. The conventional approach, such as antibiotics, has minimal effectiveness. Additionally, due to the emergence of (multiple) resistance, their use is under intense scientific and public scrutiny. Hence, there is a need for the development of alternative control techniques, with an emphasis on prevention, which is likely to be more cost-effective. In this study, a potential bacterial strain *Cytobacillus firmus* was isolated from polluted river sediment and characterized using a comprehensive range of techniques including biochemical, 16S rRNA sequencing and antibiogram assay. The pathogenicity of the bacteria was tested *in vivo* on *Labeo rohita* fingerlings found as non-pathogenic. Further, the bacteria were found to synthesize silver nanoparticles (AgNPs) using AgNO_3_ as a substrate. The obtained AgNPs were characterized by various methods, including UV–vis spectroscopy, FTIR (Fourier-transform infrared spectroscopy), and Transmission Emission Microscopy (TEM). The study found that the AgNPs were 20 nm in size on average. The antimicrobial activity of synthesized AgNPs was examined against the model freshwater pathogenic bacteria, *Edwardsiella tarda* and both the MIC (Minimum Inhibitory Concentration) and MBC (Minimum Bactericidal Concentration) were 0.156 μM, while biofilm inhibition activity was also observed at 0.156 μM. The AgNPs showed no haemolytic activity at 0.313 μM. Our findings suggest that *C. firmus* mediated bacteriogenic AgNPs modulate the activity of common pathogenic bacteria *E. tarda*. The thoroughness of our research process gives us confidence in the potential of applying AgNPs in aquaculture as a considerable strategy to control the *E. tarda* infection.

## 1 Introduction

Aquaculture is the world’s fastest-growing food-generating sector, with an average annual growth rate of 7.1% (Okeke et al., 2022). However, there have been more environmental consequences due to the rapid rise of the aquaculture industry. Significant volumes of contaminated effluent, including uneaten feed and excrement, are produced throughout production. Aquaculture releases nutrients and different inorganic and organic substances, including ammonium, phosphorus, microplastic, dissolved organic carbon, and organic matter, into the aquatic environment (Bisai et al., 2023; Felis et al., 2020; Kraemer et al., 2019). The animal is more prone to disease outbreaks because of the damaged aquatic habitat. For example, bacterial infections have rendered the aquaculture business socioeconomically and environmentally unsustainable during the last decade. Antibiotics and other conventional techniques that have been used in the past to mitigate stressful situations have had very little success. Furthermore, antibiotic residues in commercialized aquaculture products poses an additional risk to human health since it can lead to antimicrobial resistance (AMR) and toxicity, altering the natural microbiota of the human gut. Hence, the development of alternate management strategies is needed to mitigate microbial infection and provide protection to fish.

The use of nanoparticles as substitute antimicrobials has been previously suggested to prevent the growth of microbial resistance to antibiotics in aquaculture (Swain et al., 2014). One of the most essential properties of nanoparticles (NPs) is their large surface area compared to their nano size, which leads to a rise in unique characteristics and properties and boosts their interaction with other molecules (Alsamhary, 2020; Gahlawat et al., 2016; Ranjan and Jadeja, 2017). Among the most widely produced metal nanoparticles, AgNPs are the most studied nano antimicrobial agent. Previous studies revealed the ability of AgNPs against phytopathogens as well as catalyze the beverage industry (Hassanisaadi et al., 2022; Mangindaan et al., 2020; Poulose et al., 2014; Rónavári et al., 2017). AgNPs combat disease-causing bacteria via numerous pathways, allowing them to overcome bacterial resistance (Knetsch and Koole, 2011; Vance et al., 2015). The nanotoxicology of the synthesized AgNPs has already been studied by taking fish, tadpoles and various mammalian models, resulting in no significant effects on growth and other immunity and enzymatic parameters (Pattanayak et al., 2018; Scown et al., 2010; Swain et al., 2014; Tortella et al., 2020).

Although chemical and physical technologies can effectively generate pure, simple, inexpensive, well-defined nanoparticles in vast quantities, they are costly, involve complicated procedures and potentially harm the environment (Nasrollahzadeh et al., 2019). One of the approaches to environmentally friendly nanoparticle (NP) manufacturing involves using microorganisms, such as fungi and bacteria, for the synthesis of AgNPs (Mohanpuria et al., 2008; Nasrollahzadeh et al., 2019; Swain et al., 2016). Bacteria can survive in a varied range of adverse conditions, including extremes of high or low temperature, varying alkalinity or acidity levels and high salt concentrations (Li et al., 2016). Furthermore, microorganisms, in particular, are effective nano-factories for the bio-fabrication of very stable and monodispersed nanomaterials (NMs) (Ahmed et al., 2020; Noman et al., 2020).

In this study, we aimed to isolate a potential bacterium from polluted river sediment and investigate the bacteriogenic synthesis of AgNPs role in limiting the growth of freshwater fish pathogenic bacteria *Edwardsiella tarda*. Further, the bacteria were characterized by sequencing and phylogenetic analysis of the conserved region of bacterial genome 16S rRNA. The pathogenicity of the isolated bacterial strain was assessed using biochemical analysis, antibiogram test, and *in-vivo* challenge experiment. Afterward, the biosynthesized AgNPs were also characterized by using FTIR spectroscopy, UV–vis spectroscopy, and TEM imaging. The ability of AgNPs was also investigated to evaluate the antibacterial activity by disk diffusion assay, MIC and MBC against *Edwardsiella tarda*, which is responsible for severe mortality in freshwater fishes. The toxicity of the purified AgNPs was also investigated on fish RBC (red blood corpuscles).

## 2 Materials and methods

### 2.1 Isolation of microorganism

Throughout the summer season, the sediment samples were collected from a contaminated site along the Yamuna River in India (latitude 28° 37′ 44.19444″ and longitude 77° 15′ 12.14748″). The river sediment sample was screened for bacterial isolation according to the protocol used by Guan et al. (2020) with slight modifications. Briefly, the sediment samples were processed (Long et al., 2023; Sudarsan et al., 2021) and grows on the TSA plate supplemented with 5 mM of silver nitrate (AgNO_3_) was picked into a fresh TSB culture tube and cultured overnight at 28°C in tryptone soya broth (TSB) (Hi media, Mumbai, India) shaking at 120 rpm (Kumar et al., 2022). A single colony from the plate was picked into the fresh TSB media and incubated for a duration of 24 h at a temperature of 28°C while being continuously agitated at a speed of 120 revolutions per minute. The stock culture was maintained to have 40% glycerol and stored at −80°C for further use (Kumar et al., 2023a,b).

### 2.2 Identification of bacterial strain

The process of isolating bacterial DNA was conducted by using the sarkosyl method for DNA extraction (Sambrook and Russell, 2006). The primer UFF2 5′-GTTGATCATGGCTCAG-3′, and URF2 5′-GGTTCACTTGTTACGACTT-3′ (Behera et al., 2017) were used to amplify the 16S rRNA gene by using a 96-well thermal cycler PCR system 9700 (Applied Biosystem, Foster City, CA). An ABI 373xl capillary sequencer was used to do the sequencing in both the forward and reverse direction (Thermo Fisher Scientific, Foster City, United States). The contig assembly was performed using DNA baser 7.0 software, which involved aligning forward and reverse sequences. Subsequently, the resulting sequence was submitted to the National Centre for Biotechnology Information (NCBI) and deposited in the GenBank database. A phylogenetic tree was generated according to the neighbor-joining method by using MEGA 11.0 (Tamura et al., 2021), and the construction of the phylogenetic tree was performed with IToL v4 (Interactive Tree of Life) (Letunic and Bork, 2021).

### 2.3 Biochemical characterization and antibiogram assay of bacterial isolate

Gram-staining was the primary method used to determine if the isolated bacterium was Gram-negative or Gram-positive. The isolate was then biochemically characterized using the biochemical test strip (biochemical test strip, Hi-media, Mumbai, India).

The antibiotic resistance test was conducted by the prescribed methods provided by Bauer et al. (1966). The bacterial suspensions were diluted to concentrations of 10^6^ cells/mL using sterile Mueller-Hinton broth, as determined by spectrophotometer readings at a wavelength of 600 nm [CLARIOSTAR R Plus (BMG Labtech, Ortenberg, Germany)]. Subsequently, a 100 μL aliquot of the bacterial solution was uniformly dispersed onto Mueller-Hinton agar plates using a sterile spreader and five distinct antibiotic disks were aseptically positioned on each agar plate. The diameter of the zone of inhibition around the disk was measured with a caliper. The interpretation of bacterial sensitivity to the antibiotics that were tested was determined as either sensitive, intermediate, or resistant in accordance with the recommendations established by the Clinical and Laboratory Standards Institute (NCCLS, 2002).

### 2.4 Acclimatization of fish and pathogenicity assay

Organization for Economic Cooperation and Development (OECD) was followed for handling the experimental fishes. The animal use procedure for the experimental setting was approved by the Institutional Animal Ethics Committee, ICAR-CIFRI, Kolkata, India (IAEC/2022/05). Healthy fingerling stage *Labeo rohita* measuring 114.21 ± 2.16 mm in length and weighing 20.26 ± 1.02 gm were procured from a local hatchery. The fish were inspected regularly to ensure no clinical symptoms like redness, descaling, ulcer, hemorrhage, and discoloration on the fish body. The bacterial isolate was subcultured in a 15 mL culture tube (Abdos, Delhi, India) with TSB and incubated for 24 h at a temperature of 28°C. After incubation, the bacterial culture was centrifuged at 5,000 rpm for 5 min, and the supernatant was discarded. Twenty experimental fishes received an intraperitoneal injection of a bacterial suspension with a concentration of 1.2 × 10^7^ CFU/mL in a volume of 0.2 mL. The pathogenic *Aeromonas veronii* (accession No. ON346527) obtained from the Biotechnology laboratory, ICAR-CIFRI, was used as a positive control. Subsequently, the fish were kept in a fiberglass-reinforced plastic (FRP) tank and subjected to regular observations at 12 h intervals for a period of 120 h.

### 2.5 Green synthesis of AgNPs

AgNPs were produced using the methodology outlined by Saied et al., with some modifications (Essawy et al., 2021; Saied et al., 2022). In brief, 100 μL of pure bacterial culture of 1.5 × 10^8^ CFU/mL was adjusted using McFarland Standard 0.5 and was aseptically transferred to a sterile 50 mL tube containing TSB medium. The resulting mixture was then incubated in a temperature-controlled shaker incubator for 24 h, maintaining a constant temperature of 37°C and agitation at 180 rpm. Subsequently, the microbial biomass was centrifuged for 10 min at a speed of 10,000 rpm. The supernatant was collected, and the pellets containing cells were discarded and afterward subjected to filtration using a 0.22-micron syringe filter (Himedia, Mumbai, India) to obtain a supernatant free of cells. The supernatant was then mixed with AgNO_3_ (0.002 mg/mL, pH 8) (Sigma, Missouri, United States) The samples were incubated in a shaker incubator for 24 h at a temperature of 37°C, with agitation at a speed of 180 rpm, under circumstances of darkness to prevent oxidation of AgNO_3_ (Saied et al., 2022). However, TSB media with AgNO_3_ without the addition of bacterial culture was used as a negative control. Later, the solution became yellow to brown, indicating the production of AgNPs. AgNPs have been purified, and unwanted debris has been removed according to the method used by Zamanpour et al. (2021).

### 2.6 Concentration determination of AgNPs

The formula used by Kalishwaralal et al. (2010) for estimating the concentration of AgNPs was used to determine the concentration of biogenic AgNPs.

The average number of atoms per AgNPs was estimated using the formula.


(1) $$N=\frac{\pi \rho D^{3}}{6M}\;N_{A}$$

$$N=\frac{3.14\times 10.5\times {\left(20\times 10^{-7}\right)}^{3}\times 6.023\times 10^{23}}{6\times 107.868}$$

i.e., *N* = 3835335.097.

Where *N* = The number of atoms per nanoparticle, π = 3.14, ρ = density face-centered, cubic (fcc) silver’s density = 10.5 g/cm^3^, D is the average diameter of nanoparticle (20 nm = 20 × 10^−7^ cm), *M* = atomic mass of the silver = 107.868 g/mol, *N_A_* is the number of atoms per mole (Avogadro’s number = 6.023 × 10^23^ particles/mol).

The molar concentration of the synthesized AgNPs solution was estimated using the formula.


(2) $$C=\frac{N_{T}}{NVN_{A}}$$

$$C=\frac{0.12\times 6.023\times 10^{23}}{3835335.097\times 0.05\times 6.023\times 10^{23}}$$

i.e., *C* = 6.275 × 10^−7^ *M/L* = 627 nM/L.

Here, *C* = the molar concentration of the AgNPs solution. *N_T_* = the total number of silver atoms added as AgNO_3_ = 0.12 M, N is the number of atoms per nanoparticle (Calculated from the previous formula), *V* = the volume of the reaction in *L*, and *NA* is the Avogadro’s number = 6.023 × 10^23^ particles/mol.

### 2.7 Characterization of AgNPs

The characterization was conducted following the methodology previously employed by Hashem et al. (2022). The visual observation of AgNPs has been accomplished by conducting a comparative analysis of the brown-colored supernatant with the control samples. The UV–visible spectrophotometer [CLARIOSTAR R Plus (BMG Labtech, Ortenberg, Germany)] was used to detect the spectral analysis of AgNPs and analyzed in the range of 220-800 nm. Examining the morphological structure of the AgNPs involved the application of a 10 μL solution onto a carbon-coated copper grid with a mesh size of 300. The Transmission Electron Microscopy (TEM) (JEOL JEM-2100 HR, Japan) was operated at 200 kV (Hashem et al., 2022). Fourier Transform Infrared Spectroscopy (FTIR) discovers functional groups that reduce silver ions to AgNPs. The FTIR was operated in the wave number interval spanning from 500 cm^−1^ to 4,000 cm^−1^.

### 2.8 Assessment of the antibacterial activities of AgNPs

#### 2.8.1 Disk diffusion assay

The disk diffusion test was conducted by the Kirby-Bauer technique of assessing antimicrobial susceptibility (Bauer et al., 1966). The synthesized nanoparticles (0.627 μM) were impregnated into a 6 mm sterile disk (HiMedia, India) and dried for 1 h in a biosafety cabinet (Bhat et al., 2021). The quantification of the *E. tarda* bacterial culture, which had been incubated overnight, was determined to be 5 × 10^5^ CFU/mL using a 0.5 McFarland Standard. Subsequently, a volume of 100 μL of the bacterial culture was evenly distributed on an MH agar plate (Bhat et al., 2022). The impregnated disk of synthesized nanoparticle was placed on an agar plate along with a sterile disk soaked in normal saline, which was included as a negative control, and a disk containing kanamycin of 30 μg/L was used as a positive control.

#### 2.8.2 *In vitro* biofilm elimination

The biofilm assay suing synthesized nanoparticles was carried out according to the standard protocol followed by Tran et al. (2020). The optical density at 550 nm of the bacterial suspension was adjusted to a value of 0.1. 200 μL aliquots (0.1 OD at 600 nm) of the suspension were distributed in triplicate into the wells of a sterile 96-well plate (Tarsons, Kolkata, India). Later, different concentration of synthesized AgNPs was prepared by diluting with DNAse and RNAse-free water. The *E. tarda* was a positive control, while wells added with only Mueller-Hinton broth were negative controls. The plate was subjected to incubation at a temperature of 28°C, with static conditions maintained, for a duration of 24 h. To remove any non-adherent bacteria, a physiological solution (0.9% NaCl) was used to gently wash each well three times, with 300 μL of the solution being used for each wash. The wells were provided with a 200 μL volume of methanol with a purity of 99% and subjected to an incubation period of 2 h. The plate was air-dried overnight and subjected to a staining process for a duration of 20 min using 150 μL of a 0.1% crystal violet solution. Subsequently, the wells were washed with a moderate flow of tap water and allowed to air dry. Subsequently, the wells were filled with 150 μL of 95% ethanol to separate the dye that was attached to the bacterial cells adhering to the surface. After that, its absorbance was quantified at a specific wavelength of 570 nm CLARIOSTAR R Plus (BMG Labtech, Ortenberg, Germany).

#### 2.8.3 Minimum inhibitory concentration assay

A concentration of 0.627 μM of the synthesized nanoparticle was prepared according to the above-described method. The MIC of the synthesized nanoparticle is conducted according to the broth microdilution method in triplicate (Hussain Bhat et al., 2020; Wiegand et al., 2008). In brief, after centrifuging the overnight bacterial culture, the bacterial pellet was dissolved in sterile MH broth to produce a bacterial suspension equal to 0.5 McFarland standard solution (OD_625_ nm should be 0.08 - 0.13). Initially, 100 μL of 0.627 μM of synthesized nanoparticles were added in the first three rows of 96 well plates (Tarsons, India). In the remaining well of the first 3 columns (2nd–10th row), 50 μL of Mueller-Hinton (MH) broth was added, and the two-fold serial dilution was carried out with a multichannel pipette. Adjusted bacterial suspension (10^6^ CFU/mL, 50 μL) was added in the well of the first 3 columns (1st–10th row). In the 11th row, a bacterial suspension of 100 μL was added as a positive growth control, whereas in the 12th column, 100 μL of MH broth was added as a negative control. The plate was subjected to incubation at a temperature of 28°C for 16 h (Bhat et al., 2021). The MIC is described as the lowermost antimicrobial concentration that limits the apparent growth of the tested isolate, as seen with the unaided eye (De-Souza-Silva et al., 2018; Wiegand et al., 2008).

#### 2.8.4 Minimum bactericidal concentration assay

After the synthesized AgNPs’ MIC was completed, the Minimum Bactericidal Concentration (MBC) assay (performed according to Parvekar et al.) was carried out (Parvekar et al., 2020; Tandel et al., 2021). In brief, 50 μL samples were taken from the well that showed no apparent bacterial growth. These samples were then introduced into a sterile TSA agar plate and incubated for 16 h at 28°C (Hussain Bhat et al., 2020). Determining the MBC included observing bacterial presence or absence after incubation, particularly when the lowest concentration of AgNPs resulted in the eradication of 99.9% of the bacterial population (Parvekar et al., 2020).

#### 2.8.5 Hemolysis assays of the synthesized AgNPs

The hemolysis assays of the synthesized AgNPs were carried out on fish RBCs following the technique outlined by Hussain Bhat et al. (2020). Briefly, the cytotoxicity of the synthesized nanoparticle has been evaluated by detecting hemoglobin release from *Labeo rohita* RBCs spectrophotometrically at 540 nm. The experiment was repeated three times to guarantee the precision and reliability of the results, and the extent of hemolysis was determined by employing the specified equation to compute the percentage of hemolysis (H).


$$H=\frac{\mathrm{absorance}\;\mathrm{of}\;\mathrm{sample}-\mathrm{absorbance}\;\mathrm{of}\;\mathrm{blank}}{\mathrm{absorbance}\;\mathrm{of}\;0.2\%\mathrm{triton}\;X-\mathrm{absorbance}\;\mathrm{of}\;\mathrm{blank}}\times 100.$$

### 2.9 Statistical analysis

The data is provided in the form of mean ± SD (Standard Deviation). The data underwent analysis using one-way ANOVA and Tukey’s multiple comparison tests to evaluate the analysis of variance to discover significant differences. The chosen significance threshold was set at (*p* < 0.05). The statistics calculations were performed using IBM SPSS (version 25).

## 3 Results

### 3.1 Isolation and identification of the bacterial isolate

**Figure 1:**
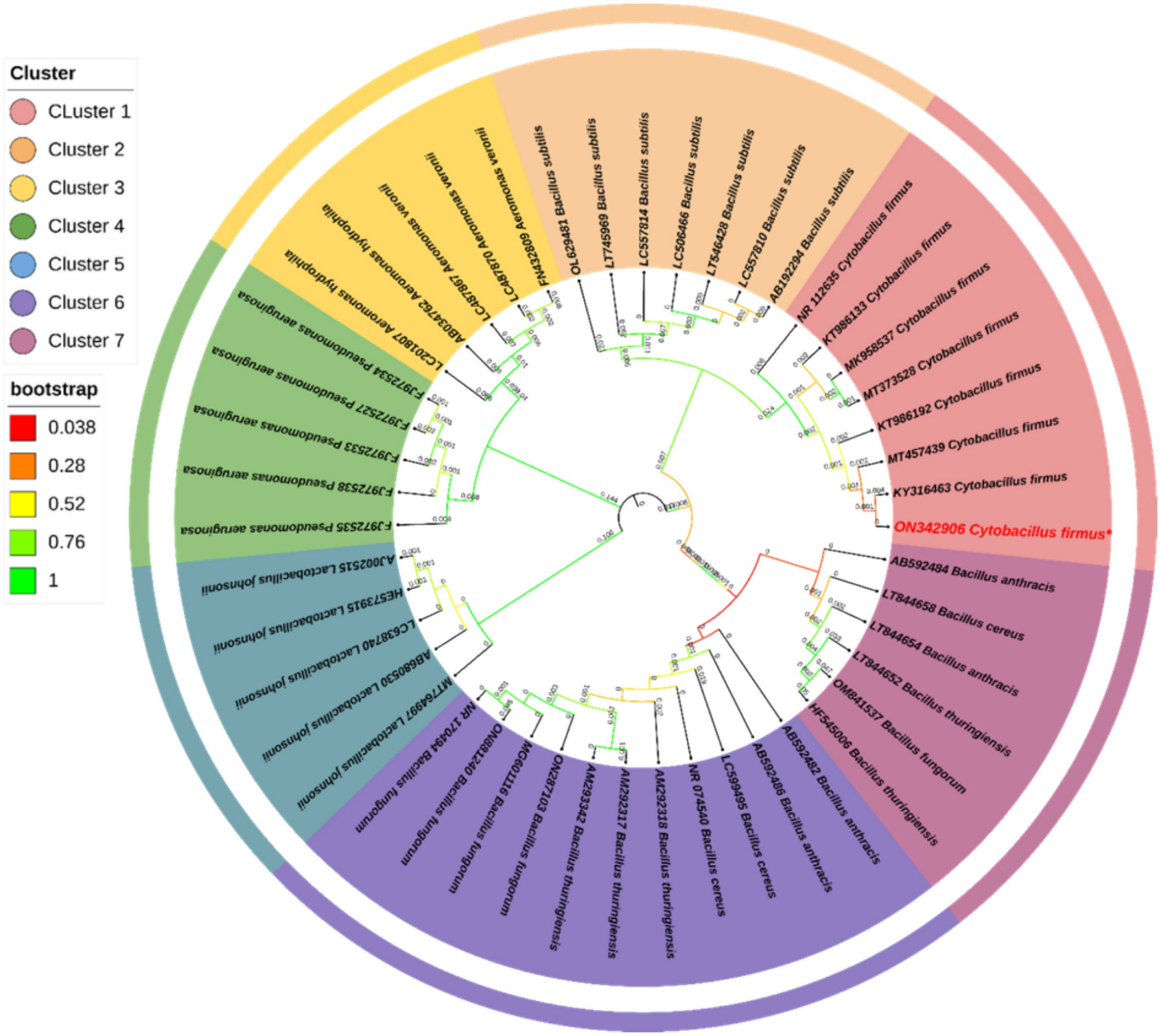
The phylogenetic study of *Cytobacillus firmus* was conducted using the 16S rRNA gene and the Neighbor-Joining (N-J) technique in MEGA 11.0. The results were visually presented in a graphical format. The legend near the phylogeny shows the bootstrap value for 1,000 replications and different clusters. The numerical value adjacent to the branches denotes the length of the branch, accurately measured to three decimal places. The Interactive Tree of Life (IToL) V4 program designed the phylogenetic tree.

The bacterial suspension obtained from the sediment sample was spread on a TSA plate with 5 mM of AgNO_3_ and showed a single type of bacterial growth that could survive in that concentration (Supplementary Figure S2). The individual colony was picked into a sterile broth medium and stored with 40% glycerol stock at −80°C for further use. The result of the PCR amplification indicates the size of the partial gene sequence was 1,445 bp (Supplementary Figure S1), and the BLAST N analysis indicated that the isolate sequence exhibited a similarity of 99.93% to *Cytobacillus firmus* (Accession No. MT457439). The results of the phylogenetic analysis indicated that the isolated strain had a tight evolutionary relationship with *Cytobacillus firmus* (Figure 1). The 16S rRNA sequencing of the bacterial strain that was obtained was deposited in the NCBI GenBank database under the accession number ON342906. The agarose gel depicting the 16S rRNA was screened (Supplementary Figure S1).

### 3.2 Biochemical characterization and antibiogram assay

The bacterial strain obtained from the sediment sample exhibited a Gram-positive phenotype upon undergoing Gram staining. The biochemical characterization revealed that the bacterial isolates were positive for Methyl red (for acid production), Nitrate reduction, Ornithine utilization, lysine utilization, Saccharose utilization, Oxidase and Trehalose utilization (Supplementary Table S1). In contrast, the test showed negative for urease activity, ONPG (β-galactosidase), phenylalanine deamination, indole, H_2_S production, citrate utilization, glucose utilization, adonitol utilization, Voges Proskauer’s (acetoin production), malonate utilization, xylose utilization, esculin hydrolysis, arabinose utilization, rhamnose utilization, cellobiose utilization, melibiose utilization, raffinose utilization and lactose utilization. The antibiogram assay revealed that the isolated strain was resistant against cefepime (CPM30) and cefixime (CFM5) (Supplementary Table S2). However, the strain was found sensitive against Kanamycin (K 30), rifampicin (RIF5), ciprofloxacin (CIP5), fosfomycin (FO200), erythromycin (E 10), netilmicin sulfate (NET30), dicloxacillin (D/C1), erythromycin (E 10), streptomycin (S25), chloramphenicol (C30), tetracycline (TE10), gentamicin (GEN 10), trimethoprim (TR5), polymyxin B (PB300), colistin (CL10), ampicillin (AMP 25), doxycycline, (DO10), imipenem (IPM10), amoxicillin (AMC 30), nalidixic acid (NA30), nitrofurantoin (NIT 200), piperacillin (PIT100/10), and tobramycin (TOB10).

### 3.3 *In-vivo* toxicity assay of *Cytobacillus firmus*

**Figure 2:**
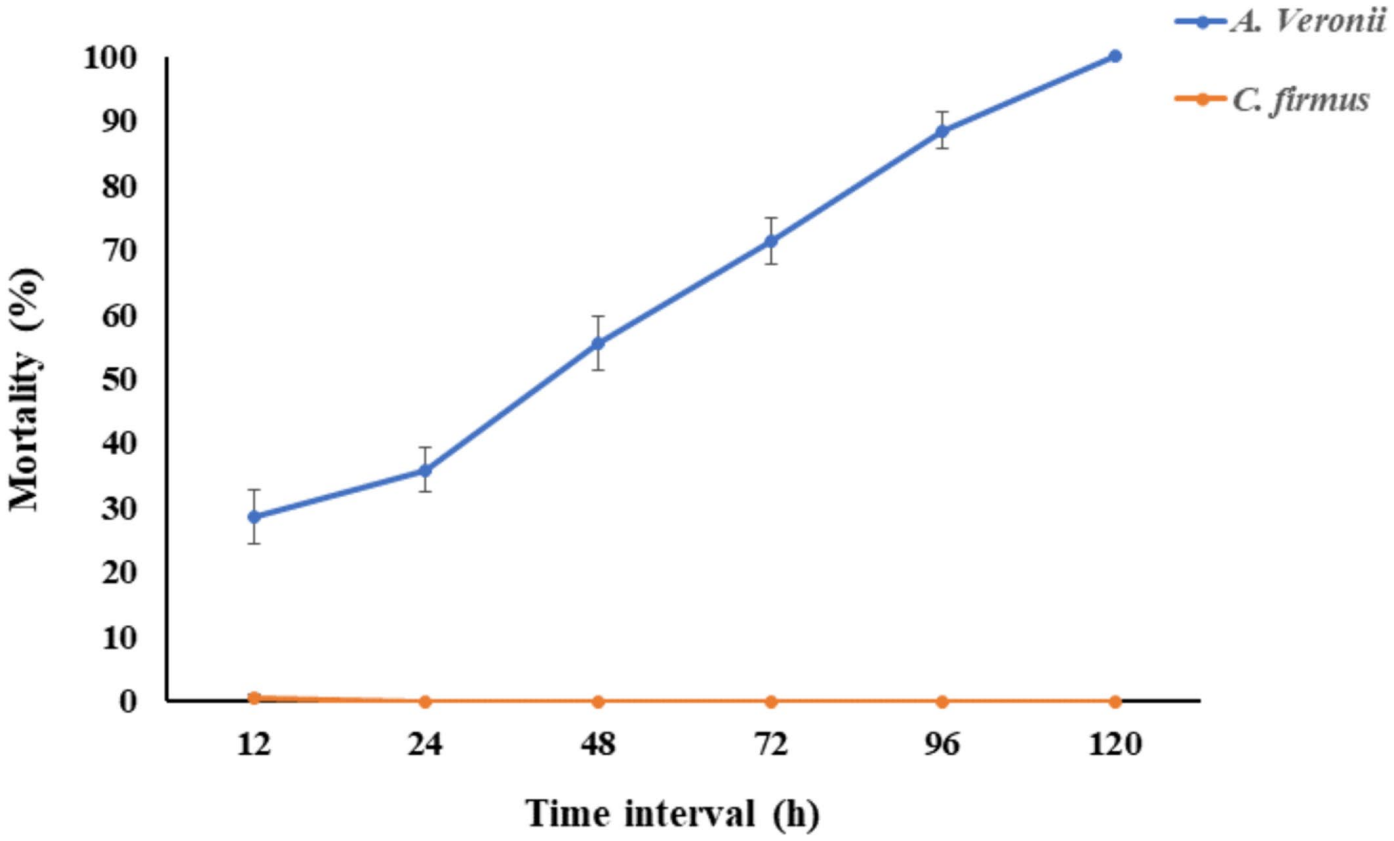
The fish mortality assay was performed to check the *pathogenicity of Cytobacillus firmus*. The Cumulative mortality percentage of *Labeo rohita* was evaluated in this figure. The orange line indicated the percentage of fish mortality when injected intraperitoneally with 200 μL of *Cytobacillus firmus* at 1.2 × 10^7^ CFU/mL. The blue line represents the percentage of cumulative mortality of fish injected with same doses of pathogenic *Aeromonas veronii*, which is the positive control. The mortality of fishes was recorded from 0 to 120 h and presented here after 12 h of interval. The data is represented as Mean ± SD, where *n* = 3 and *p* < 0.05.

The fish group challenged with bacterial doses of 1.2 × 10^7^ CFU/mL of *Cytobacillus firmus* showed no significant (*p* < 0.05) mortality within 120 h of post-infection. However, the fish group challenged with pathogenic *Aeromonas veronii* bacteria exhibited clinical symptoms like hemorrhages at the belly and resulting in a complete fatality rate of 100% seen during a 72-h period after the injection (Figure 2). Moreover, the pathogenic bacterium *Aeromonas veronii* was effectively reisolated from the liver, kidney, and blood specimens of fish exhibiting a moribund condition and identified based on colony morphology, biochemical characteristics and 16S rRNA sequencing.

### 3.4 Green biosynthesis of Ag-NP

The biosynthesis of AgNPs was observed when the cell-free supernatant of *C. firmus* was incubated with AgNO_3_ as the substrate for the synthesis of AgNPs. The visual inspection after 24 h of incubation resulted in the color of the supernatant changing to brown, while the control exhibited no significant color change after that period (Supplementary Figure S3).

### 3.5 Characterization of biosynthesize AgNP

**Figure 3:**
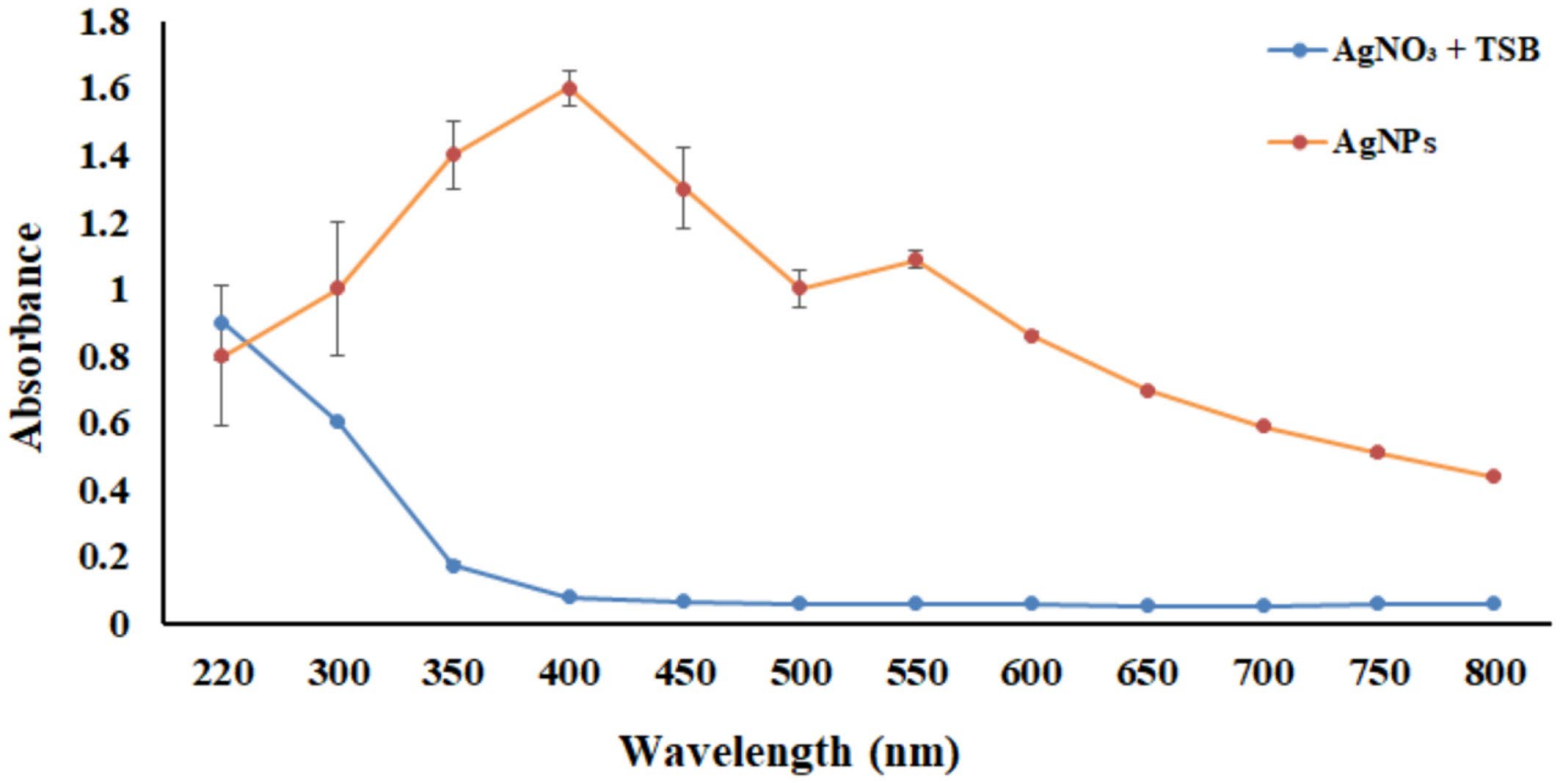
UV–vis spectrum analysis between wavelength 220–800 nm. The orange line represents the absorbance of the synthesized nanoparticles, and the blue line indicates the absorbance of only the AgNO_3_ in TSB media without bacteria.

**Figure 4:**
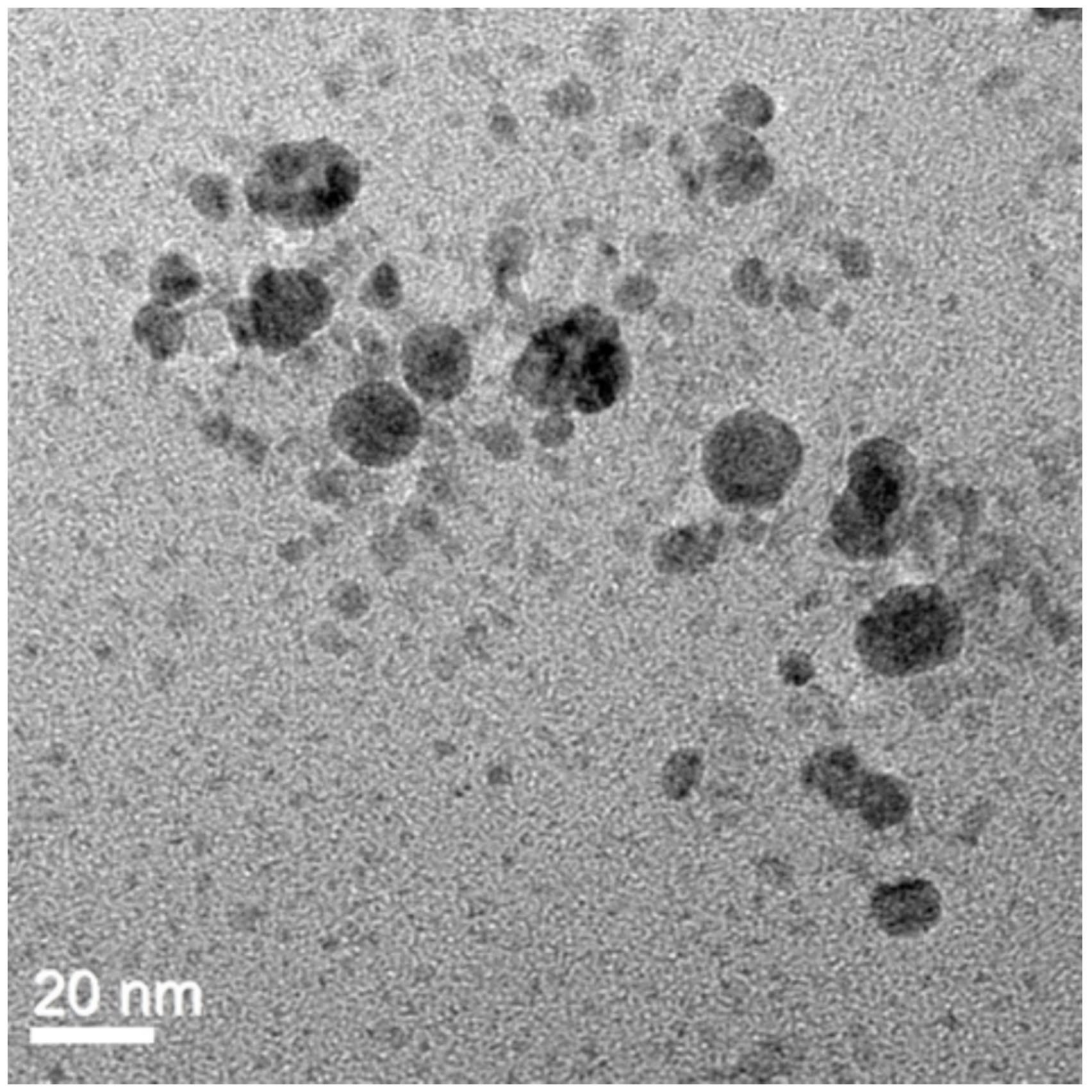
TEM image of AgNPs revealed the spherical shape of biosynthesized AgNPs with a scale bar of 20 nm.

**Figure 5:**
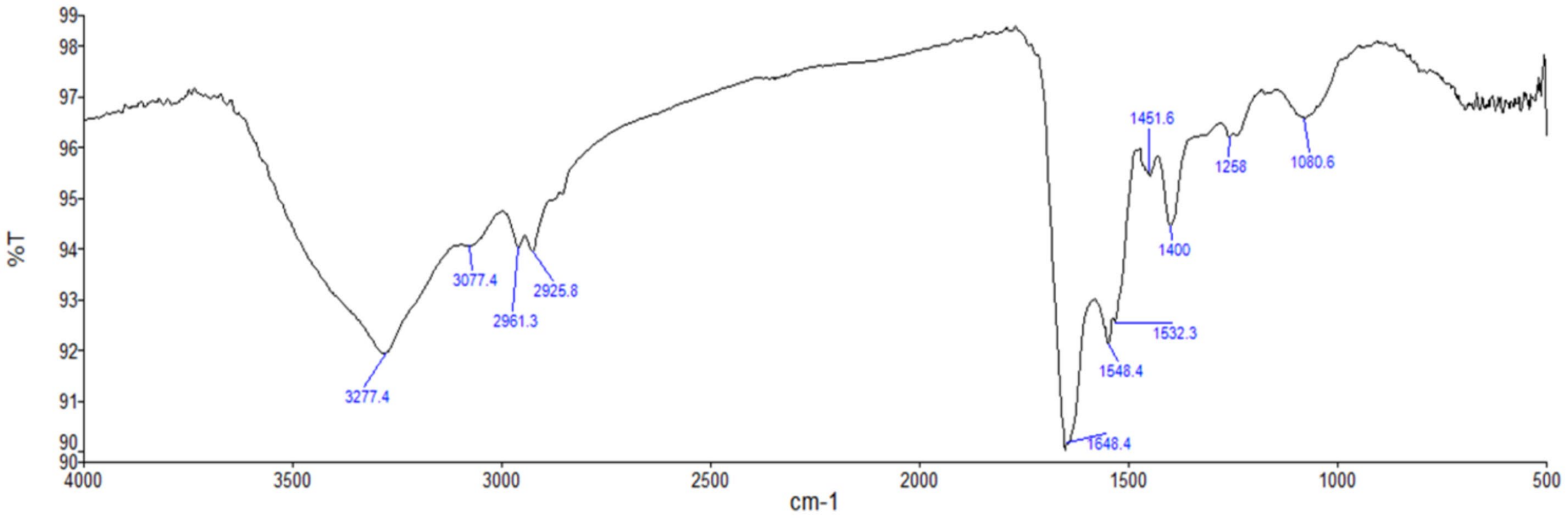
FTIR spectrum analysis of biosynthesized AgNPs. Here, the graph represents the relationship between wavenumber (cm^−1^) and transmittance percentage (%T).

UV–Vis spectrophotometer, with the absorbance of the produced nanoparticle exhibiting a peak within the wavelength range of 400–470 nm (Figure 3). The free electron of AgNPs provides surface plasmon resonance (SPR) band due to mutual vibration of electron of synthesized AgNPs in resonance with supplied light wavelength. The morphological characteristic of AgNPs was evaluated using a Transmission Electron Microscope (Figure 4). The TEM micrograph revealed the average diameter of synthesized NPs varies from 11 – 20 nm and has no sign of aggregation. Further, FTIR spectroscopy was employed to ascertain the specific functional groups that participate in the stabilization process of the produced AgNPs. The analysis revealed the absorption peak was at 3277.4 cm^−1^, 2961.3 cm^−1^, 2925.8 cm^−1^, 1648.4 cm^−1^, 1548.4 cm^−1^, 1,400 cm^−1^, 1,258 cm^−1^, 1080.6 cm^−1^. The observed peak at 3277.4 cm^−1^ may be attributed to the stretching vibrations of the O-H functional group, often seen in alcohols and phenols, as well as the N-H stretching vibrations typically present in amines. The spectral region between 2925.8 cm^−1^ and 2961.3 cm^−1^ exhibits bands attributed to the vibrational stretching of aliphatic methyl (CH_3_) and methylene (CH_2_) groups. The detected spectral peak at a wavenumber of 1648.4 cm^−1^ is most likely associated with the vibrational stretching of the carbon–oxygen (C=O) bond, typically reported in proteins. The bands seen at wavenumbers of 1,400 cm^−1^, 1,258 cm^−1^, and 1080.6 cm^−1^ may be attributed to the stretching vibrations of the carbon-nitrogen (C-N) bonds in aliphatic and aromatic amines (Figure 5).

### 3.6 Antibacterial activities of AgNPs against *Edwardsiella tarda*

**Figure 6:**
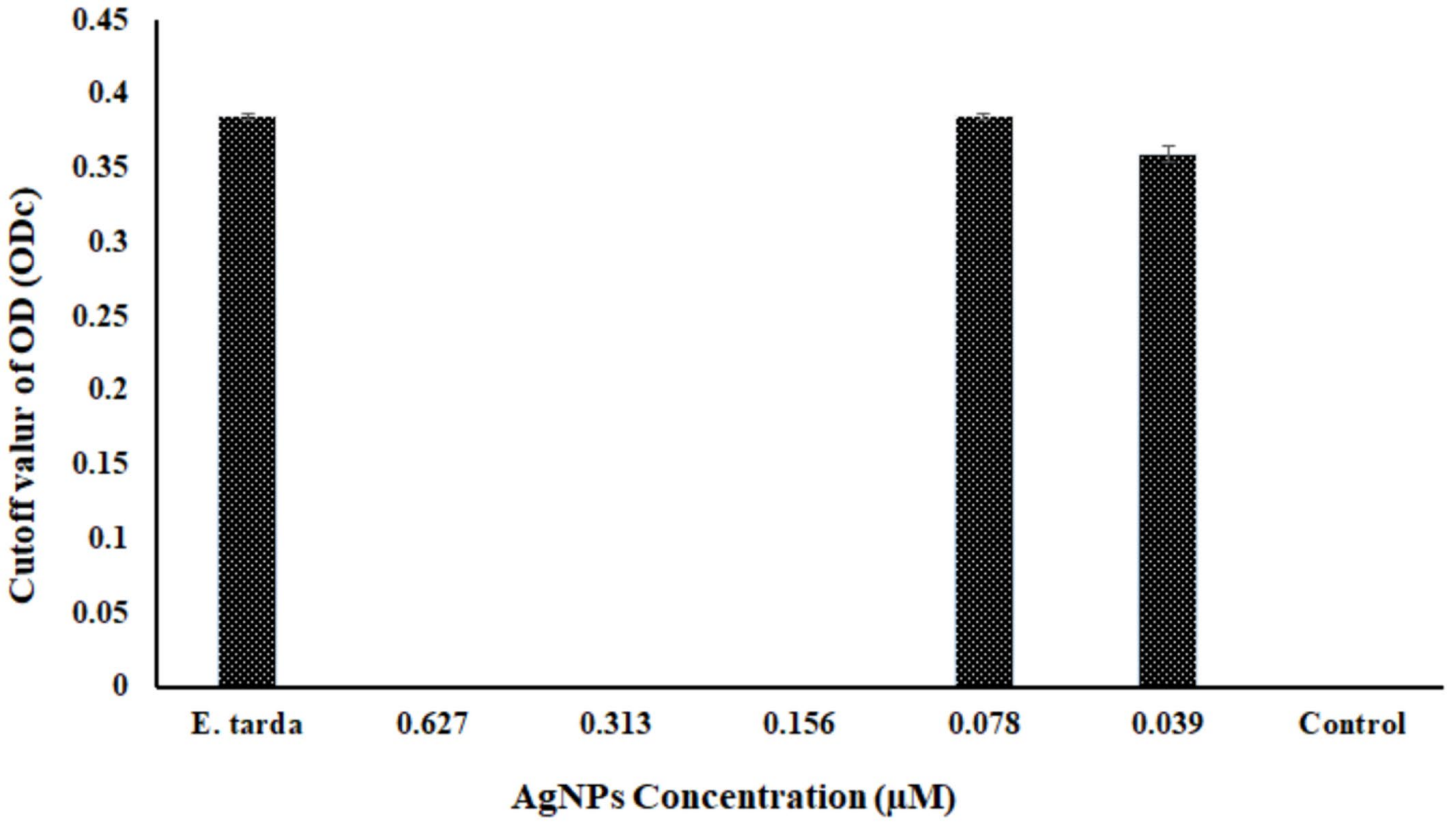
The graphical representation shows the biofilm inhibition assay at different concentrations of biosynthesized AgNPs. ODc is the cutoff OD value that is plotted against different AgNP concentrations. Here, *Edwardsiella tarda* was used as a positive control, and as a negative control, only AgNPs containing no *Edwardsiella tarda* were used; the findings are shown as Mean ± SD, *p* < 0.5.

The antibacterial efficacy of produced AgNPs against the pathogenic bacterium *E. tarda* was evaluated using the disk-diffusion technique. The results showed that at a concentration of 0.627 M, the produced AgNPs exhibited an inhibition zone of 11.5 ± 0.5 mm, whereas kanamycin of 30 μg/L as positive control showed 33.1 ± 0.7 mm. The disk impregnated with normal saline showed no zone formation (Supplementary Figure S4). The biofilm elimination assay revealed no biofilm formation in the negative control along with the top three concentrations of AgNPs (0.627 μM, 0.313 μM, and 0.156 μM). In contrast, the rest of the concentrations of the AgNPs displayed biofilm formation (Figure 6). However, in the positive control, bacteria supplemented with no AgNPs showed biofilm formation.

### 3.7 MIC and MBC values of AgNPs against *Edwardsiella tarda*

The MIC was performed in the range (0.001 μM to 0.627 μM), and the MIC of synthesized AgNPs against *E. tarda* was determined by broth microdilution method, and the AgNPs were found effective at 0.156 μM. Similarly, the Minimum Bacterial Concentration (MBC) was also effective in 0.156 μM concentration of AgNPs. Each assay was performed in triplicate where (*p* < 0.05) (Supplementary Figure S5).

### 3.8 Hemolysis assays

**Figure 7:**
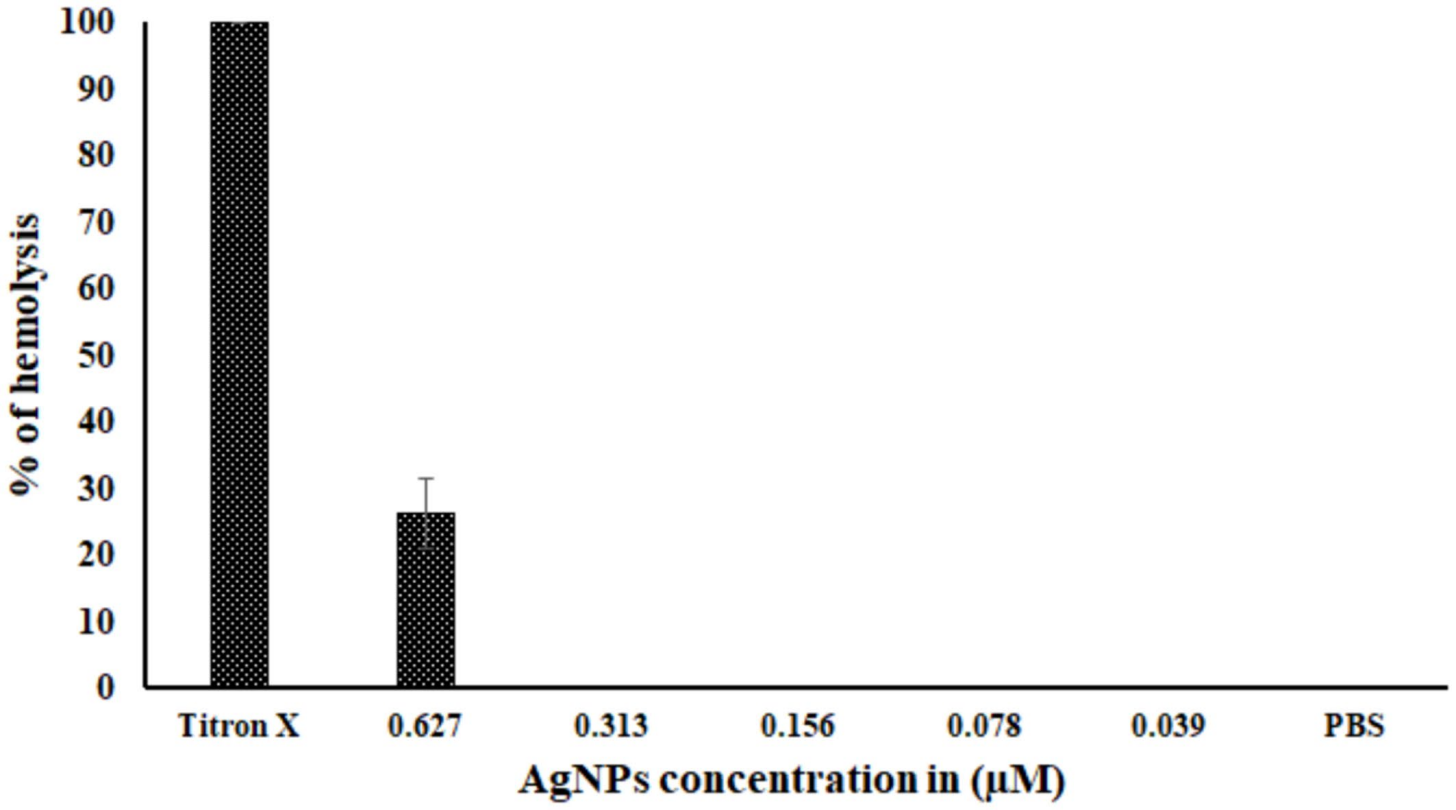
The toxicity of biosynthesized AgNPs. The graph represents the percentage of hemolysis at different concentrations of AgNPs. Titron X was used as the positive control, and PBS was used as the negative control. There were three test runs (*n* = 3), and the mean ± standard deviation (SD) indicates the proportion of hemolysis.

The hemolysis of RBCs was observed over 20% at the 0.627 μM concentration of AgNPs, whereas no cytotoxicity was observed at the other four concentrations and in negative control PBS. However, the RBCs showed 100% hemolysis for Titron X (Figure 7).

## 4 Discussion

In the last few decades, nanotechnology has significantly impacted biomedicine, diagnostics, scientific research probes, treatment options, the industrial sector, and environmental protection (Abass Sofi et al., 2022). AgNPs have emerged as a prominent subject of investigation within metal nanoparticles due to their broad application for many scientific purposes. The AgNPs exhibit toxicity against pathogens, a high surface area to volume ratio, effective cytotoxicity toward cancer cells, and their potential for catalytic applications. As a result of these characteristics, AgNPs have gained recognition and distinction in nanotechnology (Barkalina et al., 2014). Microbial-mediated synthesis of AgNPs has gained massive attention over conventional chemical and physical procedures due to the use of environmentally friendly substrates, biocompatible and the relatively more straightforward synthesis process under ambient conditions (Ameen et al., 2020; Satyanarayana Reddy et al., 2010). Previous research by Sudarsan et al. looked at the manufacture of AgNPs utilizing *Cytobacillus firmus* isolated from the stem bark of *Terminalia arjuna* (Sudarsan et al., 2021) against plant pathogenic bacteria and fungi. This motivates us to isolate potential bacteria from a source like polluted water sediment capable of producing AgNPs when supplied with AgNO_3_ as a substrate and to limit pathogenic bacteria in freshwater fishes. Furthermore, the potential effect of AgNPs against the most common freshwater fish pathogenic bacteria, *Edwardsiella tarda*, was investigated, and the AgNP concentration, which limits the bacterial activity, was also evaluated. The study highlighted that *Cytobacillus firmus-*mediated bacteriogenic AgNPs limit the growth of *Edwardsiella tarda*.

The aquatic animals living in polluted rivers possess bacteria that have unique enzymes and metabolic pathways to facilitate their chances of survival. The pollutants from refractory organics and heavy metals released from industry pollute river sediments gradually alter microbial community structure, and the bacteria gain a specific ability to survive the adverse effects (Guo et al., 2019; Zhang et al., 2020). In this study, we have isolated a microbe from sediment samples residing in polluted rivers that can produce AgNPs upon using AgNO_3_ as a substrate. Through 16S rRNA gene sequencing and subsequent phylogenetic analysis, the bacterial strain was identified as *Cytobacillus firmus*. Kabeerdass et al. demonstrated that among several microbial groups, the genus *Bacillus* has been characterized to produce AgNPs from soil (Kabeerdass et al., 2021). Our study coincides with these findings, and the isolated *Cytobacillus firmus* from a polluted river sediment sample can produce AgNPs by using AgNO_3_ as a substrate.

Biochemical reactions can provide essential data for identifying the bacterium genera in a sample (Kumar et al., 2022). Each bacteria produces a significant number of enzymes by nature, allowing them to be identified biochemically (Behera et al., 2023). For instance, Tarek et al. (2023) highlighted the importance of biochemical studies in identifying a potential protease from *Bacillus siamensis.* In this study, the recovered *Cytobacillus firmus* shows positive nitrate reductase activity, which might be responsible for the production of AgNPs upon utilization of AgNO_3_ as a substrate (Khodashenas and Ghorbani, 2016). The research findings indicate that the bacterium strain under investigation does not exhibit pathogenicity toward the *Labeo rohita* fish species and has been coinciding with previously isolated *Cytobacillus firmus* strain from the coastal region of Bhavnagar, India, revealed no sign of pathogenicity (Shinde et al., 2022).

The NADH-dependent reductase enzyme plays significant role in the reduction of Ag + ions, leading to the formation of AgNPs (Ahmad et al., 2003). It is also proposed that some enzyme and protein carbonyl groups could strengthen AgNPs by binding to the NPs surfaces (Mishra et al., 2017). In this investigation, we observed that the synthesized AgNPs underwent a color transformation from yellow to brown. Saied et al. (2022) studied the cell-free extract of *Cytobacillus firmus* for bio fabrication of AgNPs, and the AgNPs ability as an antimicrobial was studied against *Escherichia coli*, *Staphylococcus aaureus*, *Candida albicans*, *Enterococcu faecalis,* and *Pseudomonas aeruginosa.* Further, the ability to produce AgNPs has been investigated in different *Bacillus* species like *Bacillus* sp. (Kabeerdass et al., 2021; Al-Asbahi et al., 2024), *Bacillus subtilis* (Alsamhary, 2020; Satyanarayana Reddy et al., 2010), *Bacillus licheniformis* (Tandel et al., 2021)*, Cytobacilllus firmus* (Sudarsan et al., 2021), *B. pumilus* (Esmail et al., 2022).

LSPR (localized surface plasmon resonance) is commonly regarded as feedback on NP formation (Mie et al., 2014; Mulvaney, 1996; Shukla and Iravani, 2017). The surface plasmon resonance band of AgNPs may be seen using a UV spectrum ranging from 400 to 470 nanometers (Jyoti et al., 2016; Zamanpour et al., 2021). The present study indicate that the presence of AgNPs may be detected by the prominent surface plasmon resonance peak seen at around 410 nm. Additionally, the presence of a single intense band indicates that the biosynthesized AgNPs possess a spherical shape and are of small size (Zamanpour et al., 2021). Nanoparticles have a wide range of morphology: spherical, rod-like, decahedral, triangular, and platelet forms (Mock et al., 2002). TEM analysis revealed that the particle seems spherical in shape without aggregation and that the sizes range from 10 to 25 nm. A similar kind of data was obtained by (Saied et al., 2022) who synthesized the AgNPs. Analysis showed that the nanoparticle was spherical in shape and the size ranged from 30 to 60 nm. The identification of possible biomolecules responsible for the reduction of silver ions, efficient capping, and stability of the produced AgNPs has been accomplished by the utilization of FTIR spectroscopy. The prior investigation using FTIR spectroscopy, identified a peak at 3390.24 cm^−1^, which was determined to be related to the O-H stretching group of phenol and alcohol and the band seen at a wavenumber of 1666.2 cm^−1^ may be attributed to the interplay between the stretching vibrations of the N–H bonds and the amide I band of proteins (Saied et al., 2022). In this study, the peak observed at 3339.54 cm^−1^, 2961.3 cm^−1^, 2925.8 cm^−1^, 1648.4 cm^−1^, 1548.4 cm^−1^, 1,400 cm^−1^, 1,258 cm^−1^, 1080.6 cm^−1^ revealed the chemical groups linked to the AgNPs. Furthermore, apart from the electrostatic attraction shown by negatively charged carboxylate groups in enzymes, the presence of free amine groups and cysteine residues in proteins might also contribute to the binding of nanoparticles to proteins (Mie et al., 2014).

Edwardsiellosis caused by *Edwardsiella tarda* was reported from diverse host ranges and geographical distribution, possesses key virulence factors essential in bacterial survival, and causes disease in wild and farmed fish species (Park et al., 2012). The work by Shaalan et al. (2017) studied the chemically synthesized AgNPs role in antimicrobial activity against *E. tarda*. In the present study, the antibacterial activity by disk diffusion assay revealed the bacteriogenic synthesized AgNPs show a clear zone at 0.627 μM concentration. The precise mechanism of AgNPs antibacterial activity is yet to explore. However, the most convincing theory is silver ions generated from AgNPs can electrostatically adhere to negatively charged proteins in the bacterial cell membrane, causing protein structural disintegration of the cell membrane and resulting in cell death (Lin and Xing, 2007). The previous study has shown the ability of AgNPs to limit the biofilm-forming phenomenon of *Pseudomonas aeruginosa* and *Escherichia coli* (Zamanpour et al., 2021), *Pseudomonas aeruginosa* and *Staphyloccus epidermidis* (Kalishwaralal et al., 2010). Simultaneously, this investigation demonstrated that the antibiofilm efficacy of the produced AgNPs exhibited a concentration-dependent relationship. Specifically, the biofilm development by *Edwardsiella tarda* was suppressed entirely at concentrations equal to or exceeding 0.156 μM.

The minimum inhibition concentration (MIC) and Minimum Bactericidal concentration (MBC) of AgNPs against pathogenic *Edwardsiella tarda* were found in parallel where synthesized AgNPs MIC and MBC against *E. coli* were 31.25 μg/mL (Zamanpour et al., 2021). This study reports the cytotoxicity study on *Labeo rohita* RBCs. The concentration of AgNPs that triggered lysis of 30% of RBCs was 0.627 μM.

## 5 Conclusion

The study was designed to isolate a potential bacterium (*Cytobacillus firmus*) from the sediment samples of a polluted river that can synthesize AgNPs utilizing AgNO_3_ as a substrate. The secreted AgNPs were characterized by UV–vis spectroscopy, FTIR analysis, and TEM imaging techniques. Our study concluded the use of *C. firmus* in the biogenic production of AgNPs is therefore determined to be an economical, sustainable, and easy process that eliminates the risks associated with potentially toxic reducing/capping agents. Therefore, using bacteriogenic AgNPs as an antimicrobial to limit the most potential freshwater bacterial pathogen, *Edwardsiella tarda,* in aquaculture might be suggested as an alternative. In the future, it will allow other researchers to better understand the microbiota of sediments from a polluted river.

## Data Availability

The original contributions presented in the study are included in the article/Supplementary material, further inquiries can be directed to the corresponding author.
