# Supplementary material for: Biogenic synthesis of silver nanoparticle by Cytobacillus firmus isolated from the river sediment with potential antimicrobial properties against Edwardsiella tarda
Source: Front Microbiol. 2024 Aug 30;15:1416411. doi: 10.3389/fmicb.2024.1416411 (PMC11392742; doi:10.3389/fmicb.2024.1416411)
Supplement: Supplementary file 1 [file Data_Sheet_1.docx]

**Supporting information’s**


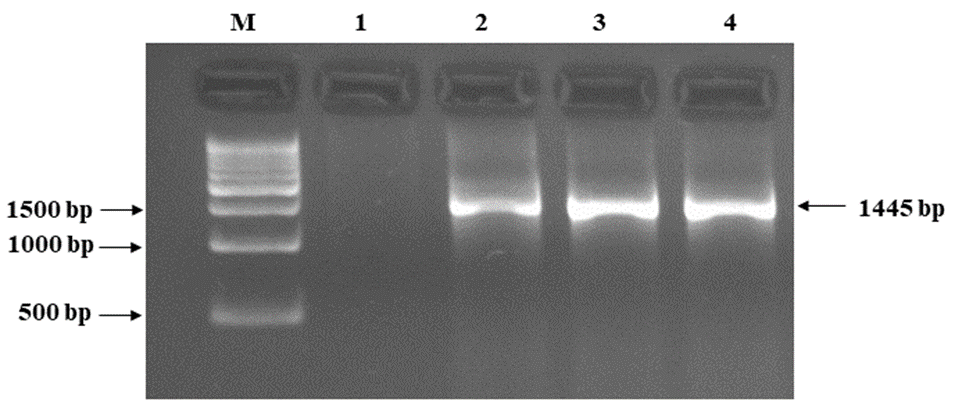


**Figure S1.** Gel image of amplification of 16S rRNA gene, shows M-500 bp ladder, Lane 1 denotes negative control and Lane 2, 3 and 4 denotes amplicon of 1445 bp of 16S rRNA gene**.**


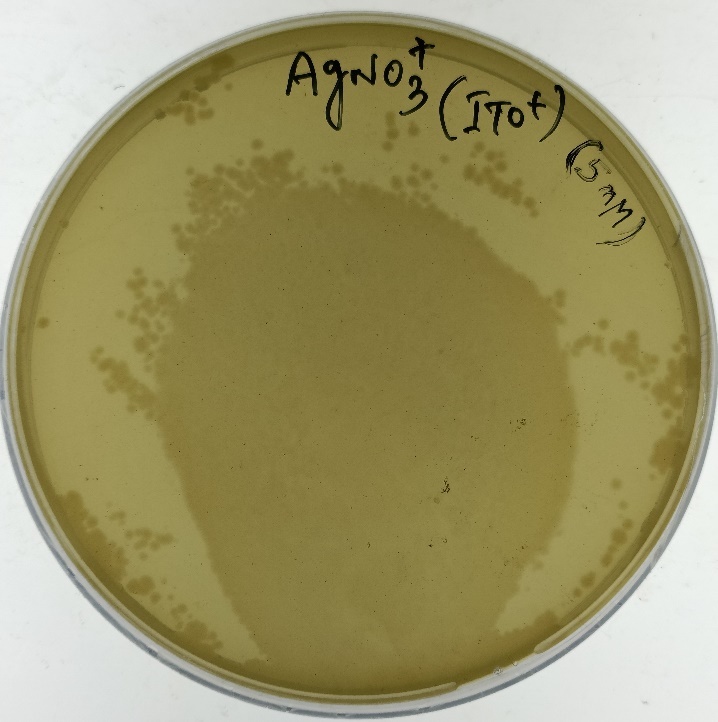


**Figure S2.** *Cytobacillus firmus* strain colony on TSA agar media supplemented with 5 mM AgNO_3_ by spread plate method.


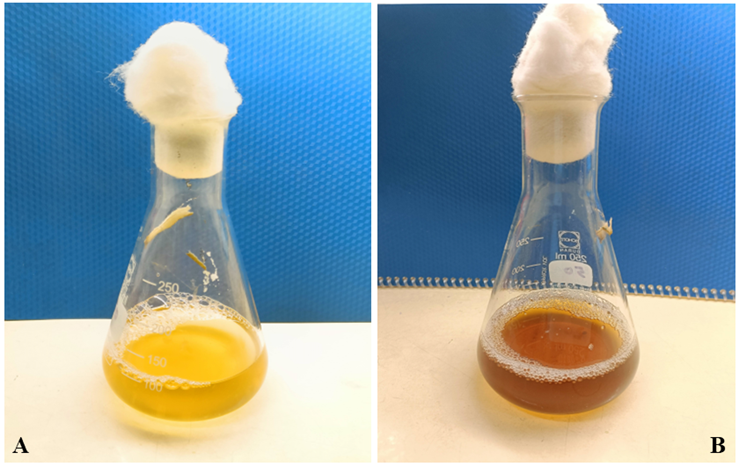


**Figure S3.** Pictorial representation of *C. firmus* mediates nanoparticle synthesis (A) Picture of the conical flask containing the *Cytobacillus firmus* biomass in TSB media without AgNO_3_ after 72 h of post incubation (B) flask containing *Cytobacillus firmus* biomass with 12 mM of AgNO_3_ after 72 h of post-incubation.


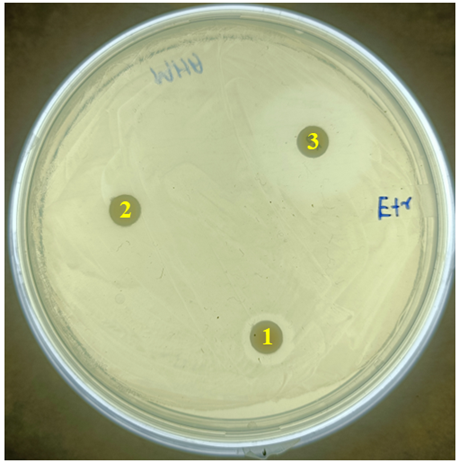


**Figure S4.** Antimicrobial activity of AgNPs at 0.627 µM against *Edwardsiella tarda* by disk diffusion assay (1) AgNPs (2) Negative control (3) kanamycin of 30 mg/L.


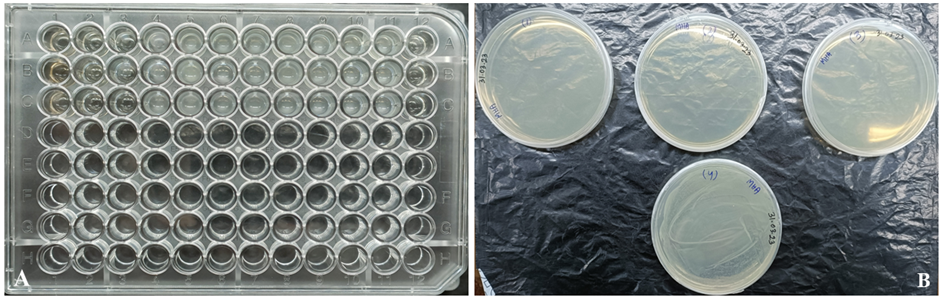


**Figure S5.** (A) MIC assay at 96-well plate reveals no bacterial growth at top three concentration of AgNPs. (B) The MBC was represented as least concentration of AgNPs which shows no bacterial growth. Both the assay was performed in triplicate (n = 3).

**Table S1.** Biochemical characterizations of the bacterial isolates.

| **Sl. no** | **Test** | **Result** |
| --- | --- | --- |
|  | ONPG (β-galactosidase) | - |
|  | Lysine utlization | + |
|  | Ornithine utlization | + |
|  | Urease activity | - |
|  | Phenylalanine Deamination | - |
|  | Nitrate reduction | + |
|  | H_2_S production | - |
|  | Citrate utlization | - |
|  | Voges Proskauer’s (acetoin production) | - |
|  | Acid production | + |
|  | Deamination of tryptophan | - |
|  | Malonate utilization | - |
|  | Esculin hydrolysis | - |
|  | Arabinose utilization | - |
|  | Xylose utilization | - |
|  | Adonitol utilization | - |
|  | Rhamnose utilization | - |
|  | Cellobiose utilization | - |
|  | Melibiose utilization | - |
|  | Saccharose utilization | + |
|  | Raffinose utilization | - |
|  | Trehalose utilization | + |
|  | Glucose utilization | - |
|  | Lactose utilization | - |
|  | Cytochrome oxidase production | + |

The isolates in biochemical assay exhibited different activity are expressed as positive (+) and negative (-).

Table S2 Antibiotics susceptibilities of Cytobacillus firmus isolated from Yamuna River sediment

| **SL. No** | **Antimicrobial** | **Disc concentration (µg)** | **Mean zone diameter (mm)** | **Sensitivity** |
| --- | --- | --- | --- | --- |
|  | Kanamycin | 30 | 31.33 | S |
|  | Rifampicin | 5 | 17.66 | S |
|  | Fosfomycin | 200 | 13.33 | S |
|  | Cefixime | 5 | 42.33 | S |
|  | Cefepime | 30 | 0 | R |
|  | Ciprofloxacin | 5 | 0 | R |
|  | Ofloxacin | 2 | 37.33 | S |
|  | Erythromycin | 10 | 22 | S |
|  | Netilmicin | 30 | 33.33 | S |
|  | Dicloxacillin | 1 | 19 | S |
|  | Streptomycin | 25 | 31 | S |
|  | Chloramphenicol | 30 | 23.66 | S |
|  | Tetracycline | 10 | 37.66 | S |
|  | Gentamicin | 10 | 14.33 | S |
|  | Trimethoprim | 5 | 20 | S |
|  | Polymyxin B | 300 | 20 | S |
|  | Colistin | 10 | 17.33 | S |
|  | Ampicillin | 25 | 17 | S |
|  | Doxycycline | 10 | 39.66 | S |
|  | Imipenem | 10 | 38.33 | S |
|  | Amoxicillin | 30 | 31.66 | S |
|  | Nalidixic acid | 30 | 30.33 | S |
|  | Nitrofurantoin | 200 | 18 | S |
|  | Piperacillin/Tazobactam | (100/10) | 18.33 | S |
|  | Tobramycin | 10 | 30 | S |

‘R’ denotes resistance and ‘S’ denotes susceptibility
